# A possible explanation for resistance in schizophrenia

**DOI:** 10.1192/j.eurpsy.2025.1192

**Published:** 2025-08-26

**Authors:** S. Laabidi, O. Laabidi, A. Touiti, A. Aissa, N. Bram

**Affiliations:** 1 forensic psychiatry; 2 psychiatry A, Razi hospital, manouba, Tunisia

## Abstract

**Introduction:**

Arachnoid cysts are intra-arachnoid space-occupying brain lesions, typically of a benign, congenital nature.Such cysts are quite rare, accounting for only 1% of all lesions in the intracranial space. In most cases, they are diagnosed accidentally by neuroimaging.Treatment-resistant schizophrenia (TRS) has a high burden both for patients and healthcare services. There is a need to identify treatment resistance earlier in the course of the illness, in order that effective treatment can be offered promptly. Recently, the co-occurrence of arachnoid cysts and schizophrenia has captured the popular attention about possible relevancy.

**Objectives:**

Through a case report and a review of the literature, we hypothesize that arachnoid cyst is the cause of resistance in a patient with treatment-resistant schizophrenia.

**Methods:**

Starting from a case report, we conducted a literature review on “PubMed”, using key words “arachnoid cyst, arachnoid cyst and psychosis”, “arachnoid cyst and treatment-resistant schizophrenia”,

**Results:**

We present a 47-year-old who is single and unemployed. His past psychiatric history revealed a diagnosis of schizophrenia, having been admitted several times in different inpatient psychiatric wards. In the psychiatric examination, the presence of auditory hallucinations, dissociated thinking, and predominantly negative symptoms was observed. His symptoms showed only minimal responsiveness.He was diagnosed with TRS owing to the inadequate response to two sequential antipsychotic trials (with adequate dose, duration, and adherence).Our evaluation of TRS began with a thorough review of the patient’s psychiatric and treatment history. All nonpsychiatric causes, including untreated medical problems, that may contribute to ongoing psychotic symptoms have been ruled out. Physical examination and blood tests were unrevealing.Electroencephalography showed no signs of seizure activity. Following the evaluation process, a head CT scan showed a left paramedian cystic lesion at the level of the pineal gland. A cerebral MRI was performed in order to get a more detailed image. It confirmed the nature of the lesion and revealed the existence of an arachnoid cyst about 2.5 cm × 3.5 cm × 2.0 cm in size, centered on the quadrigeminal cistern with triventricular dilatation. This neurological tumor didn’t require neurosurgery.

**Conclusions:**

Our case emphasises the importance of considering an organic cause like any space-occupying lesion in the brain (an arachnoid cyst in our case) for the induction of psychopathological symptoms, even those of treatment-resistant schizophrenia, which represents a major clinical challenge. This also underlines the interest of neuroimaging in the initial workup and supports the hypothesis of psychosis as a global network.

**Disclosure of Interest:**

None Declared

